# A Clutter-Analysis-Based STAP for Moving FOD Detection on Runways

**DOI:** 10.3390/s19030549

**Published:** 2019-01-29

**Authors:** Xiaoqi Yang, Kai Huo, Xinyu Zhang, Weidong Jiang, Yong Chen

**Affiliations:** 1 Graduate School, National University of Defense Technology, Changsha 410073, China; yangxiaoqinudt@sina.com; 2 College of Electronic Sciences and Technology, National University of Defense Technology, Changsha 410073, China; zhangxinyu9011@163.com (X.Z.); jwd2232@vip.163.com (W.J.); 3 Science and Technology on Electromagnetic Scattering Laboratory, Beijing 100854, China; yonche@163.com

**Keywords:** FOD, inhomogeneous clutter, IID sample decoupling, scattering cell division, STAP

## Abstract

Security risks and economic losses of civil aviation caused by Foreign Object Debris (FOD) have increased rapidly. Synthetic Aperture Radars (SARs) with high resolutions potentially have the capability to detect FODs on the runways, but the target echo is hard to be distinguished from strong clutter. This paper proposes a clutter-analysis-based Space-time Adaptive Processing (STAP) method in order to obtain effective clutter suppression and moving FOD indication, under inhomogeneous clutter background. Specifically, we first divide the radar coverage into equal scattering cells in the rectangular coordinates system rather than the polar ones. We then measure normalized RCSs within the X-band and employ the acquired results to modify the parameters of traditional models. Finally, we describe the clutter expressions as responses of the scattering cells in space and time domain to obtain the theoretical clutter covariance. Experimental results at 10 GHz show that FODs with a reflection higher than −30 dBsm can be effectively detected by a Linear Constraint Minimum Variance (LCMV) filter in azimuth when the noise is −60 dBm. It is also validated to indicate a −40 dBsm target in Doppler. Our approach can obtain effective clutter suppression 60dB deeper than the training-sample-coupled STAP under the same conditions.

## 1. Introduction

The background of this research is the multi-death crash of Concorde Air France in 2000, which was caused by a piece of debris on the taxiway and evinces the need to detect Foreign Object Debris (FOD) on runways. FODs may lacerate aircraft tires or wear engines [1] during taking off and landing. According to statistics from Insight SRIT, the authoritative analysis company in UK, over 66% of airport emergencies are related to FOD [2]. It has become the second most common threat to aviation security after bird hit.

The International Civil Aviation Organization stipulates explicitly that at least four-time inspections per day must be ensured to the runway. While manual inspection can only guarantee safety for 1% of the flights, the automated FOD detection systems could provide nearly 100% effective inspections for all flights [3].

In existing systems [4,5,6,7], the radar and Electro-Optical (EO) hybrid devices are most commonly utilized. But EO sensors will be greatly weakened in inclement weathers [8,9]. In comparison, radars can provide all-time and all-weather inspection to runways [10]. Wide band millimeter-wave radars have high enough resolutions to detect small pieces of metal, stones, concrete or even plastics on runways. For instance, COBRBA-220 [11], the ultra-wide-band system, could reach a resolution of 1.8 cm. The 77 GHz FOD detection radar [12] developed under the cooperation between Japan and France showed signal attenuation less than 0.18 dB as well as high sensitivity to −20 dBsm objects. In recent years, performances of some other radars working around 70 GHz [13], 76.5 GHz [14], 78 GHz [15,16], 96 GHz [17], even 110 GHz [18] have been successively validated by test data in controlled conditions (e.g., wave form, polarization, and antenna gain).

Although stationary FOD detections have been well developed by Constant False Alarm Rate (CFAR) algorithms [19,20,21,22], the radar-based FOD surveillance is still challenged by finding debris in different motion states (e.g., rolling small screws, wind-driven plastic bags [23,24] and invading wildlife [1] (p. 1), especially in clutter conditions [3].

Space-time Adaptive Processing (STAP) [25,26] has been maturely utilized in Synthetic Aperture Radar (SAR) to suppress ground clutter and indicate motive targets [27]. The same technique can potentially be utilized to design space-time filters to detect motive FOD in strong clutter, if exact clutter covariance estimation is obtained. STAP requires that the number of training samples [28] must have more than double Degrees of Freedom (DOFs) and be Independent Identically Distributed (IID) with the detected samples. However, these requirements are rarely satisfied in practice, which makes the STAP performance limited [29].

A series of methods have been proposed to release the IID constraint, and thus to accelerate computation and convergence. The most representative and widely used are the reduced-dimension and the reduced-rank STAPs [30,31,32], which operate on the basis of matrix transformations. The Sparse-Recovery (SR) STAP technique [33,34] has attracted great attention because it can reduce computation significantly in the case of insufficient training samples by making utilization of clutter sparsity in the space-time plane. By employing environment knowledge, Knowledge Aided- (KA-) STAPs are proposed with significant superiority, prominent value, and wide prospect to radar intellectualization. By utilizing prior information in algorithms directly, Bayesian filtering [35,36] and data pre-whitening STAPs [37,38] are investigated but the performance is influenced by the mismatch between prior knowledge and time-varying environment. Some other ideas have concerned environment sensing for intelligent sample selection [39,40] to analyze rather than estimate the clutter covariance by samples. But these STAPs demand exact backscattering coefficients and high resolution cells, with the support of real scene topography [41,42], digital elevation model data [43], hyper-spectral remote sensing images [44] and so on.

Based on the above analysis, we propose a clutter-analysis-based STAP for motive FOD detection in a familiar environment, which decouple from IID training samples to estimate clutter covariance. The prior knowledge could be easily obtained from the SAR observations, the high-resolution visible spectrum images, or the airport construction drawings [45] (pp. 4–7).

The rest of this paper is organized as follows: in Section 2, the radar coverage is divided into scattering cells based on the geometric model in *xOy* coordinates. In Section 3, the space-time clutter is deduced and addressed according to the parameter-modified scattering model. Section 4 concerns the filter design in the space-time domain. Experiments and discussions are overviewed in Section 5 that support the conclusions drawn in Section 6.

## 2. Scattering Cells

A novel scattering cell division method is introduced in this section as the foundation of clutter analysis. The geometric model of an airport is constructed at first to determine the radar covering area. Note that the polar coordinates are not applicable in this case. Therefore, we divide the scene into scattering cells in *xOy* coordinates for exact backscattering coefficients.

### 2.1. Geometric Model of Scene

Federal Aviation Administration has regulated FOD detection systems referring to installation, operation, maintenance, and renewal. Most equipment should be operated at a distance of greater than 50 m away from the runway and taxiway [41] and with a height of less than the safety limit (usually two meters) [41,45]. Below is the model depicting the geometrical relationships between the SAR and the scene.

**Figure 1:**
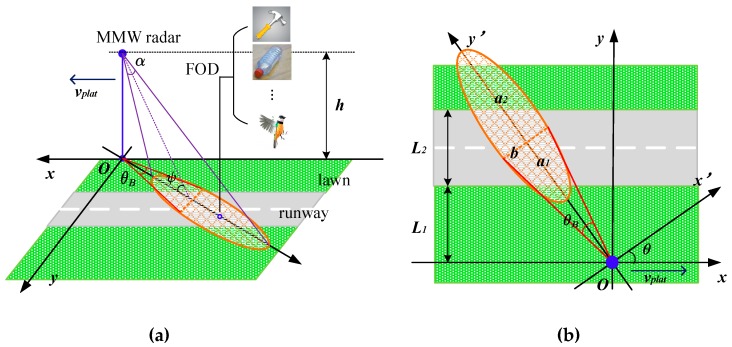
(**a**) Geometrical model of foreign object debris (FOD) detection; (**b**) The vertical view of (a).

As depicted in Figure 1a, the rectangular coordinates are better suited to the straight runway, where the origin is at the location of the platform and *x* axis is parallel to the runway. The side looking SAR, equipped with a Uniform Linear Array (ULA), is deployed on a platform *L*_1_ away from the *L*_2_-width runway and travels at a velocity of *ν_plat_* towards the positive direction of the *x* axis. Note that the maximum radar range is much larger than the platform height *h*, which causes a very small grazing angle *ψ*.

To simplify the analysis, we suppose that the radiation energy is concentrated within the main beam whose width is *θ_B_* in azimuth and *α* in elevation. The orange area in Figure 1b highlights the area covered by the SAR beam. It could be divided into two semi-ellipses sharing the same short axis *b*, but they have different long axes denoted by *a*_1_ and *a*_2_. With the beam scanning, the coordinates could be transformed from *xOy* to *x’Oy’* by *θ* between the beam and *x* axis:

(1) $$\{\begin{array}{c} x^{\prime }=x\cos\theta +y\sin\theta \\ y^{\prime }=y\cos\theta +x\sin\theta \end{array}$$

The center (*x*_0_’, 0) is expressed as following:

(2) $$\{\begin{array}{c} \frac{{\left(x^{\prime }-x_{0}{}^{\prime }\right)}^{2}}{a_{1}{}^{2}}+\frac{{y^{\prime }}^{2}}{b^{2}}=1,x^{\prime }\leq \frac{h}{\tan\psi }\\ \frac{{\left(x^{\prime }-x_{0}{}^{\prime }\right)}^{2}}{a_{2}{}^{2}}+\frac{{y^{\prime }}^{2}}{b^{2}}=1,x^{\prime }>\frac{h}{\tan\psi } \end{array}$$

*a*_1_, *a*_2_, *b* and *x*_0_’ are given in Equation (3).

(3) $$\{\begin{array}{c} a_{1}=\frac{h}{\tan\psi }-\frac{h}{\tan\left(\psi +\alpha /2\right)}\approx \frac{h}{\psi }-\frac{h}{\psi +\alpha /2}\\ a_{2}=\frac{h}{\tan\left(\psi -\alpha /2\right)}-\frac{h}{\tan\psi }\approx \frac{h}{\psi -\alpha /2}-\frac{h}{\psi }\\ b=h\tan\left(\theta_{B}/2\right)/\sin\psi \\ x_{0}{}^{\prime }=h/\tan\psi \approx h/\psi \end{array}$$

where *ψ* is extremely narrow, which makes the approximate equality relations hold. Combining all of the above, Equation (2) is rewritten as

(4) $$\{\begin{array}{c} \frac{{\left(x\cos\theta +y\sin\theta -h/\psi \right)}^{2}}{{\left[h/\psi -h/\left(\psi +\alpha /2\right)\right]}^{2}}+\frac{{\left(y\cos\theta -x\sin\theta \right)}^{2}}{{\left[h\tan\left(\theta_{B}/2\right)/\psi \right]}^{2}}=1,x\cos\theta +y\sin\theta \leq h/\psi \\ \frac{{\left(x\cos\theta +y\sin\theta -h/\psi \right)}^{2}}{{\left[h/\left(\psi -\alpha /2\right)-h/\psi \right]}^{2}}+\frac{{\left(y\cos\theta -x\sin\theta \right)}^{2}}{{\left[h\tan\left(\theta_{B}/2\right)/\psi \right]}^{2}}=1,x\cos\theta +y\sin\theta >h/\psi \end{array}$$

Equation (4) defines the area where clutter is generated, acting as the foundation of the following analysis. Without loss of generality, we focus on θ=π/2 to analyze for more simple expressions.

### 2.2. Scattering Cell Dividing

Traditional STAPs perform well only in a homogeneous clutter background [44,46] and are usually investigated in polar coordinates. However, various terrains sharing the same range may result in different distributed clutter in airports. In this case, clutter covariance estimation by training samples will be not reliable.

**Figure 2:**
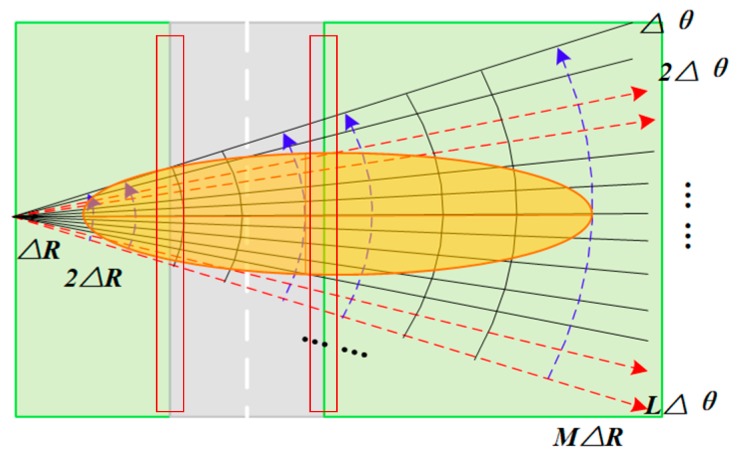
Dividing the scene into resolution cells in polar system.

Ground clutter is reflected by the statistical echoes of resolution cells, thus the cell area and backscattering coefficients are both required to calculate the RCS. The common range-azimuth dividing is depicted in Figure 2. The resolutions represented by ΔR and Δθ can be obtained according to the parameters of the transmitting wave. But those cells at the border between different scattering surfaces, such as runways and lawns, are hard to provide exact backscattering coefficients for clutter analysis. Therefore, we consider discussing the *xOy* system by dividing the radar covering area into several equal grids, which is expected to be more applicable to the straight runways.

**Figure 3:**
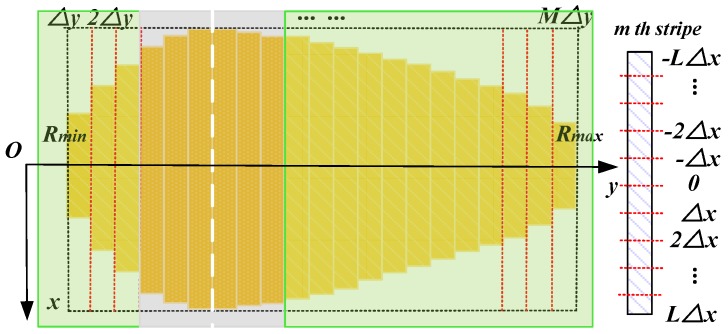
Dividing the radar coverage into several grids sized Δx×Δy.

As is shown in Figure 3, we assume the beam exists in the dashed square. We divide the radar coverage into *M* equal stripes. The width of each stripe is $\Delta y=\left(R_{\max}-R_{\min}\right)/M$. Each stripe contains *2L* grids sized Δx×Δy. Thus, every stripe is 2LΔx in length. We define the grid centers as $\left(x_{c}\left(l\right),y_{c}\left(m\right),\right)$, where l=−L,⋯,0,⋯,L, m=1,⋯,M. Notice that the cell area ΔS(l,m)=ΔxΔy is independent of l and m. These grids are defined as the clutter scattering cells in this paper. We consider that each cell is isotropic where the backscatter coefficient keeps constant.

## 3. Ground Clutter Deduction

According to the electromagnetic scattering theory, the ground clutter can be reasonably modeled by considering all scattering cells even in inhomogeneous clutter environments. In this section, first the classical Kulemin model is modified for low-grazing backscatter coefficients by the measured normalized RCS data in X-band and then the space-time coupled clutter is further deduced and investigated specifically for analysis and synthesis purposes.

### 3.1. Test-based Kulemin Model

At extremely narrow grazing angles (less than 5 degrees), the backscattering coefficient (RCS per unit area) acquirement is a problem in practical cases since the ground-clutter echo is hardly collected by the antennas. A series of empirical models covering almost 0–90 degrees are presented to depict the clutter statistics characteristic. Among them, the Kulemin model shows applicability at 3–100 GHz and low grazing angles less than 30 degrees, containing only three parameters determined by averaging the experiment data under several surface conditions (including humidity, roughness and vegetation types) [47] (pp. 17–20). Thus, it is usually preferred for simple expression and wide range applications to achieve backscattering coefficients of the concrete runways and surrounding lawns as a preliminary clutter estimation. The model is given in detail as [41]

(5) $$\xi^{0}\left(\mathrm{dB}\right)=A_{1}+A_{2}log\left(\psi /20\right)+A_{3}log\left(f_{c}/10\right)$$

where the carrier frequency $f_{c}$ and the grazing angle ψ are evaluated in GHz and degree respectively. However, the echo is too small to be statistically significant when ψ is very close to zero, which is seldom taken into consideration. Obtained by generalization of different cases, $A_{1}$ to $A_{3}$ are chosen according to Table 1:

Based on the experimental evidence, this model could describe the statistical trend of $\xi^{0}$ ideally but hardly complete accurate clutter modeling in certain conditions.

Even with a lack of measuring data at small grazing, test results in higher elevation cases have the potential to modify $A_{1}$ to $A_{3}$ by LS fitting (the derivation and calculation details are given in Appendix A), so as to approach the low-grazing situations. More specifically, the low-grazing $\xi^{0}$ could be directly calculated when $f_{c}$ is put into the modified model.

**Figure 4:**
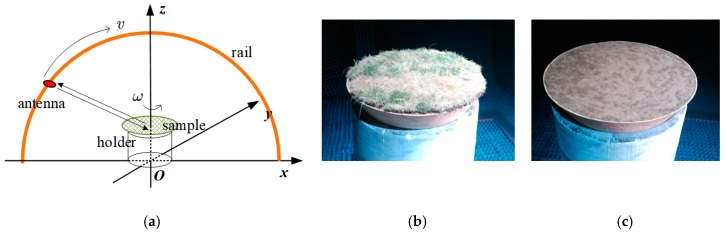
(**a**) The schematic diagram of measurement set-up; (**b**) the grass sample; (**c**) the concrete sample.

The measuring data have been gathered in our labs. Figure 4a shows the experimental set-up. The typical ground samples (see Figure 4b,c), located on a uniformly rotating holder, are produced into round shapes to minimize the effect of sample configurations. The grazing angle is controlled by an antenna scanning along the arc rail and the azimuth is determined with the holder rotating. Measured RCS data is collected in different azimuth under a certain grazing angle. In addition, sync pulses are employed to synchronize continuous wave transmission and data acquisition.

**Figure 5:**
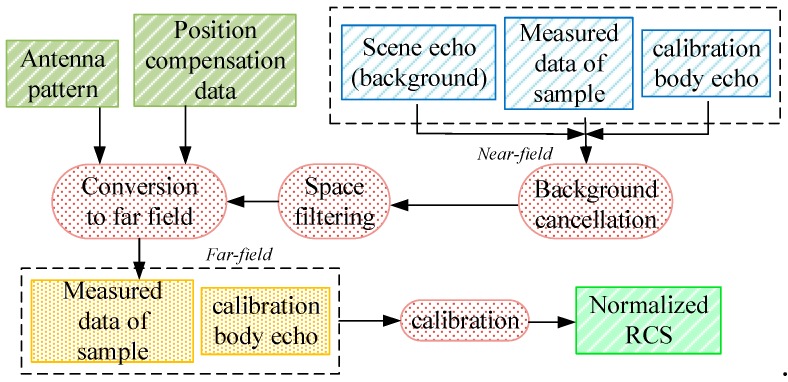
The flow diagram of measured data processing procedures.

The diagram in Figure 5 explains the measured data processing procedures, mainly involving background cancellation, transformation between near and far-field, as well as calibration. In detail, a standard metal ball with known RCS value has been used as the calibration body in the external calibration experiment at first. Considering the experiment space, the samples are measured at near field. The dotted-line box indicates the echoes received by antenna. Through background cancellation, the echo power is improved by a space filter, and converted to far-field with the support of antenna pattern and position compensation data. The measured sample RCSs are gathered in different azimuth angles and normalized by employing the calibration factor of the standard body. Hence normalized RCSs are obtained by averaging these data in different measuring conditions, which is equal to the backscatter coefficients in numeral.

### 3.2. Space-time Coupled Clutter

With the SAR travelling at $v_{plat}$ speed, the Doppler ranges from $-f_{d\;\max}$ to $f_{d\;\max}$ with the relative motions between scene and radar, where $f_{d\;\max}=2v_{plat}/\lambda$ (λ represents the wave length). In another word, the frequency band of ground clutter spreads to $2f_{d\;\max}$ wide [46,48]. It is reflected by the ground clutter distributing across the space-time domain, which is known as space-time coupling. Referring to Section 2.1, we deduce the space frequency $f_{sc}$ and Doppler $f_{dc}$ of ground clutter in rectangular coordinates as

(6) $$\begin{array}{c} f_{sc}\left(l,m\right)=\left(d/\lambda \right)\left[x_{c}\left(l\right)/\sqrt{x_{c}{\left(l\right)}^{2}+y_{c}{\left(m\right)}^{2}+h^{2}}\right]\\ f_{dc}\left(l,m\right)=\left(2v_{plat}T_{r}/\lambda \right)\left[x_{c}\left(l\right)/\sqrt{x_{c}{\left(l\right)}^{2}+y_{c}{\left(m\right)}^{2}+h^{2}}\right] \end{array}$$

where $T_{r}$ is the Pulse Repetition Interval (PRI) and d denotes the element spacing in ULA.

Drawing on the traditional STAPs, $\beta =2v_{plat}T_{r}/d$ is employed to describe the linearity between $f_{sc}$ and $f_{dc}$ given as $f_{dc}\left(l,m\right)=\beta f_{sc}\left(l,m\right)$ when θ=π/2 [49] (pp. 17–19). In case of *N* elements and *K* pulses in a Coherent Process Interval (CPI), ground clutter in each cell is

(7) $$\begin{array}{c} c_{n,k}\left(l,m\right)=\rho_{ck}\left(l,m\right)\exp\left\{2\pi j\left[nf_{sc}\left(l,m\right)+kf_{dc}\left(l,m\right)\right]\right\},\\ n=1,\cdots ,N,\;k=1,\cdots ,K \end{array}$$

$\rho_{ck}\left(l,m\right)$ satisfies $\xi_{c}\left(l,m\right)=E\left\{{\left|\rho_{ck}\left(l,m\right)\right|}^{2}\right\}$ where $\xi_{c}\left(l,m\right)=\xi^{0}\left(l,m\right)\Delta S\left(l,m\right)$ is introduced to account the clutter intensity. Obviously, $\xi_{c}\left(l,m\right)$ is almost decided by $\xi^{0}\left(l,m\right)$ in the cell at $\left(x_{c}\left(l\right),y_{c}\left(m\right)\right)$. Hence the steering vectors are expressed as

(8) $$\begin{array}{c} v_{\mathrm{sc}}={\left\{\exp\left[2\pi jf_{sc}\left(l,m\right)\right],\cdots ,\exp\left[2\pi Njf_{sc}\left(l,m\right)\right]\right\}}^{T}\\ v_{\mathrm{dc}}={\left\{\exp\left[2\pi jf_{dc}\left(l,m\right)\right],\cdots ,\exp\left[2\pi Kjf_{dc}\left(l,m\right)\right]\right\}}^{T} \end{array}$$

Referring to Section 2.2, we define the clutter in each cell as $c{\left(l,m\right)}_{NK\times 1}$

(9) $$c\left(l,m\right)={\left[c_{1,1}\left(l,m\right),\cdots ,c_{1,K}\left(l,m\right),\cdots ,c_{n,K}\left(l,m\right),\cdots ,c_{N,K}\left(l,m\right)\right]}^{T}$$

which could also be expressed in the form of a Kronecker product:

(10) $$c\left(l,m\right)=\rho_{ck}\left(l,m\right)\left[v_{\mathrm{sc}}\left(l,m\right)⊗v_{\mathrm{dc}}\left(l,m\right)\right]$$

In practical operation, the clutter covariance matrix could be estimated by

(11) $${\hat{R}}_{c}=\frac{1}{LM}\overset{M}{\underset{m=1}{\sum }}\overset{L}{\underset{l=1}{\sum }}c\left(l,m\right)c^{H}\left(l,m\right)$$

to approach $R_{c}=E\left[\mathbb{C}{\mathbb{C}}^{H}\right]$ where

(12) $$\mathbb{C}=\left[\begin{array}{ccc} c\left(1,1\right) & \cdots & c\left(1,M\right)\\ \vdots & \ddots & \vdots \\ c\left(L,1\right) & \cdots & c\left(L,M\right) \end{array}\right]$$

We notice that the estimation decouples from the IID training samples. In other words, it focuses on modelling the clutter with the aid of topography knowledge of runways to obtain reliable ${\hat{R}}_{c}$, which offers an unprecedented idea as the preliminary of STAP.

## 4. Clutter-Analysis-Based STAP

Clutter is required to be suppressed in the space-time domain by effective filtering. Traditional STAPs perform well under homogeneous clutter background by estimating clutter covariance from real echo directly. Notice that clutter echo from some range gates, which contains only one isotropic scattering surface (as Figure 2 depicts), is considered homogeneous, thus enough IID training samples could be gathered. Scattering cell division is not required considering the increasing computation. As for those range gates involving both lawn and concrete runways, we utilize the presented STAP to achieve exact ${\hat{R}}_{c}$ for effective filter weight solution, considering the limited performance of some previous methods. Two approaches could be selected according to practical clutter cases, combining the characteristics and advantages of them.

**Figure 6:**
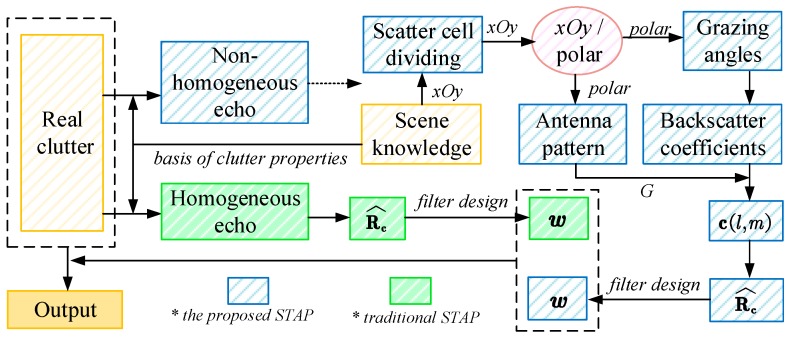
The flow diagram of traditional and proposed Space-time Adaptive Processing (STAPs) application.

According to all discussion above, the flow chart shows the processing steps involving the proposed STAP as well as traditional method for FOD detection:

See Figure 6, the clutter properties (homogeneous or not) could be known according to the corresponding range gates. To the homogeneous clutter echo, generated by lawn surface only, we employ traditional STAPs to estimate clutter covariance matrix from real IID samples directly (indicated by the green blocks in the flow diagram). Aided by the scene knowledge and a parameter-modified scattering model, we utilize the clutter-estimation-based STAP for effective suppression to the non-homogeneous clutter at those range gates involving runways and lawns, through scene-knowledge-aided division of scattering cells in *xOy* coordinates, transformation to polar coordinates (illustrated by pink), back-scattering coefficient acquirement based on the known grazing angles, and clutter echo deduction with the antenna pattern *G*, and ${\hat{R}}_{c}$ estimation, as depicted by the blue blocks. Note that the dotted arrow in the figure implies the proposed STAP is real-clutter-decoupling.

Before solving the filter weight, the space-time coupled echoes of FOD items are first deduced. For general analysis, we consider all possible targets as one or more scattering points. Taking a point target moving at $v_{FOD}$ in radial direction in the cell at $\left(x_{t},y_{t}\right)$ as the example, the space frequency and Doppler are written as

(13) $$\begin{array}{c} f_{st}=\left(d/\lambda \right)\left(x_{t}/\sqrt{x_{t}{}^{2}+y_{t}{}^{2}+h^{2}}\right)\\ f_{dt}=\left(2v_{plat}T_{r}/\lambda \right)\left(x_{t}/\sqrt{x_{t}{}^{2}+y_{t}{}^{2}+h^{2}}\right)+2v_{FOD}T_{r}/\lambda \end{array}$$

$v_{\mathrm{st}}$ and $v_{\mathrm{dt}}$ in space and time domains are expressed as [49]

(14) $$\begin{array}{c} v_{\mathrm{st}}={\left[\exp\left(2\pi jf_{st}\right),\cdots ,\exp\left(2\pi Njf_{st}\right)\right]}^{T}\\ v_{\mathrm{dt}}={\left[\exp\left(2\pi jf_{dt}\right),\cdots ,\exp\left(2\pi Kjf_{dt}\right)\right]}^{T} \end{array}$$

Thus, the target echo is as the following equation shows [49]

(15) $$s_{t}=\sqrt{\xi_{t}}\left(v_{\mathrm{st}}⊗v_{\mathrm{dt}}\right)=\rho_{t}\left(v_{\mathrm{st}}⊗v_{\mathrm{dt}}\right)$$

in the condition that the scattering intensity $\xi_{t}$ and the amplitude $\rho_{t}$ satisfies $\rho_{t}=\sqrt{\xi_{t}}$. The optimal weight vector obeying Linearly Constrained Minimum Variance (LCMV) is:

(16) $$w_{opt}=\mu {\left\{E\left[\left(s_{t}+c+n\right){\left(s_{t}+c+n\right)}^{H}\right]\right\}}^{-1}\left(v_{\mathrm{st}}⊗v_{\mathrm{dt}}\right)=\mu {\left(R_{s}+R_{c}+R_{n}\right)}^{-1}(v_{\mathrm{st}}⊗\;v_{\mathrm{dt}})=\mu R_{x}{}^{-1}\left(v_{\mathrm{st}}⊗v_{\mathrm{dt}}\right)=\mu R_{x}{}^{-1}v_{t}$$

which is known as the Wiener solution where μ = ${\left(v_{t}{}^{H}R_{x}{}^{-1}v_{t}\right)}^{-1}$ [50]. $R_{x}{}^{\prime }=R_{x}-R_{s}$ is preferable than $R_{x}$ in practice to avoid signal cancellation [49] (pp. 22,23). Thus, the target Doppler and azimuth are both acquired according to $\hat{w_{opt}=}\mu {\left(R_{s}+{\hat{R}}_{c}+R_{n}\right)}^{-1}\left(v_{\mathrm{st}}⊗v_{\mathrm{dt}}\right)$.

## 5. Experiments and Discussion

To evaluate the models, deductions, and conclusions above, experiments are presented according to the data setting in Section 5.1. The simulation results of the scattering cell division, the fitting results of Kulemin model, the MV spectrum of clutter, as well as the LCMV space-time filter are discussed in Section 5.2.

### 5.1. Dataset

**Figure 7:**
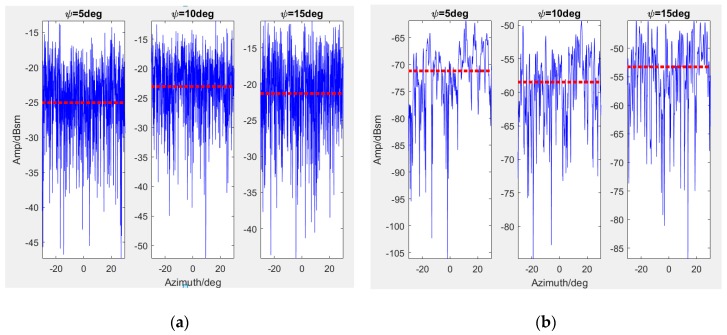
Measured data and the statistical average values about the backscattering coefficients from Science and Technology on Electromagnetic Scattering Laboratory (**a**) Dataset 1 (**b**) Dataset 2 ($f_{c}=10\;\mathrm{GHz}$).

As is presented in Figure 4, the grass and concrete samples are produced and measured on a rotating holder under the conditions illustrated in Table 2. Dataset 1 is the result of the grass sample and Dataset 2 is that of the concrete sample when the polarization is VV. The test data are displayed in Figure 7, when the grazing angle are controlled as 5, 10, and 15 degrees. Note that Figure 7a only shows a part of the data under −30 to 30 degrees.

The blue lines represent the data amplitude while the red dotted lines denote the average values of different azimuth cases at certain frequencies. Hence the data would be employed to modify Kulemin models by LS fitting. Referring to Section 3.1, the modified models at 10 GHz are firstly calculated and expressed as the implements to analyze scattering properties in low grazing angles.

### 5.2. Simulations and Discussion

**Figure 8:**
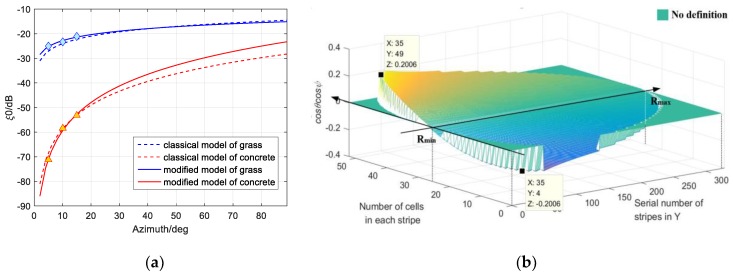
(**a**) Comparison between traditional Kulemin model and the data fitting results; (**b**) cosθcosψ of each cell ranges from −0.2006 to 0.2006, which constrains $v_{\mathrm{st}}$, $v_{\mathrm{dt}},$ $v_{\mathrm{sc}},$ and $v_{\mathrm{dc}}$.

**Figure 9:**
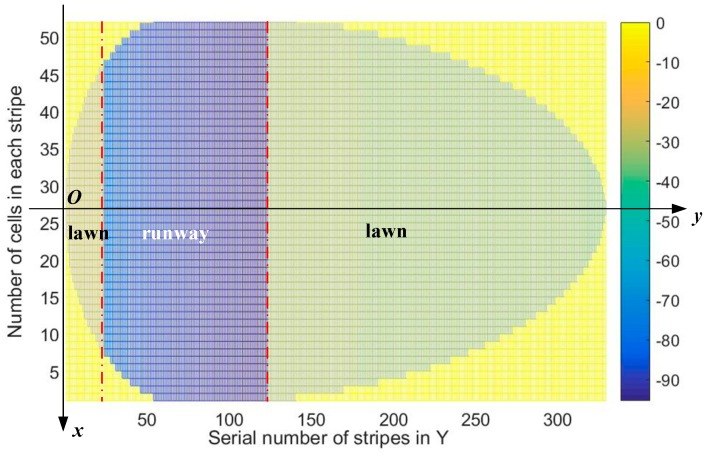
Scattering properties within the radar coverage, on basis of the parameter-modified models in Figure 8a when the cell size is 0.5 m × 0.5 m.

Table 3 provides the simulation setting:

LS fitting results based on Dataset 1 and 2 are given in Figure 8a compared with the Kulemin models. They are considered reliable for the similar trends with the classical models. The coefficients of concrete are at least 36 dB less than those of the grass surface when the grazing angle is very small (≤10 degrees). Note that cosθcosψ also plays as the foundation of $v_{\mathrm{st}}$, $v_{\mathrm{dt}},$ $v_{\mathrm{sc}},$ and $v_{\mathrm{dc}}$, therefore we calculate $\cos\theta \cos\psi \left(l,m\right)=x_{c}\left(l\right)/\sqrt{x_{c}{\left(l\right)}^{2}+y_{c}{\left(m\right)}^{2}+h^{2}}$ of every cell covered by the SAR in Figure 8b to acquire the corresponding $\xi^{0}$ when Δx=Δy=0.5m. In this case, the grazing angles of cells increase from −0.2006 to 0.2006 and the scattering properties are displayed in Figure 9.

**Figure 10:**
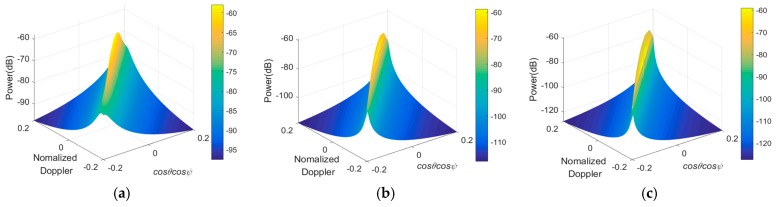

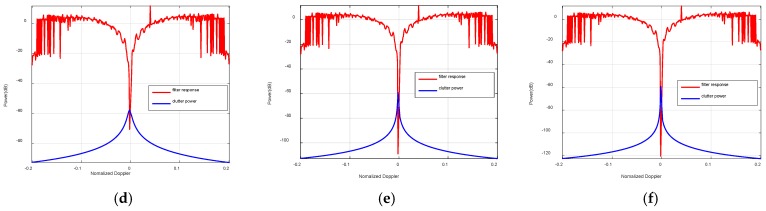
The minimum variance (MV) power spectrum of clutter and noise shows space-time coupling indicated briefly by the ridge-shaped field when the noise power is (**a**) −60 dBm; (**b**) −80 dBm; (**c**) −90 dBm; the optimal response obeying linearly constrained minimum variance (LCMV) when the noise power is (**d**) −60 dBm; (**e**) −80 dBm; and (**f**) −90 dBm.

With the side-looking SAR travelling, clutter Doppler is greatly expanded. FOD detection will be challenged because the target is severely fuzzed in the Doppler domain. As the basis of clutter suppression, the MV spectrum is ideal in evaluating the clutter visually and qualitatively. According to Section 3.2, we investigate the MV spectrum of clutter and noise on the space-time plane in Figure 10a–c when the noise power is −60 dBm, −80 dBm and −90 dBm. The ridge-shaped field reflects $f_{dc}\left(l,m\right)=\beta f_{sc}\left(l,m\right)$ only when θ=π/2, known as the main clutter field, indicates where the clutter power mainly focuses. Red lines in Figure 10d–f provide the corresponding clutter suppression when utilizing a LCMV space-time filter.

Obviously, the space-time coupled clutter is significantly suppressed to nearly −70 dB, −110 dB, and −120 dB, which is ideal referring to the corresponding red lines, which illustrate the power at clutter ridges achieving about 40 dB, 60 dB, and 70 dB higher than that on the other field of space-time plane. Taking a LCMV filter as the example, it works well for the following reasons: the power of the desired signal can be kept and the variance of the filter output is minimized as well. In fact, some filters obeying the other criteria (e.g., MSNR, MMSE) could also reach similar performances.

**Figure 11:**
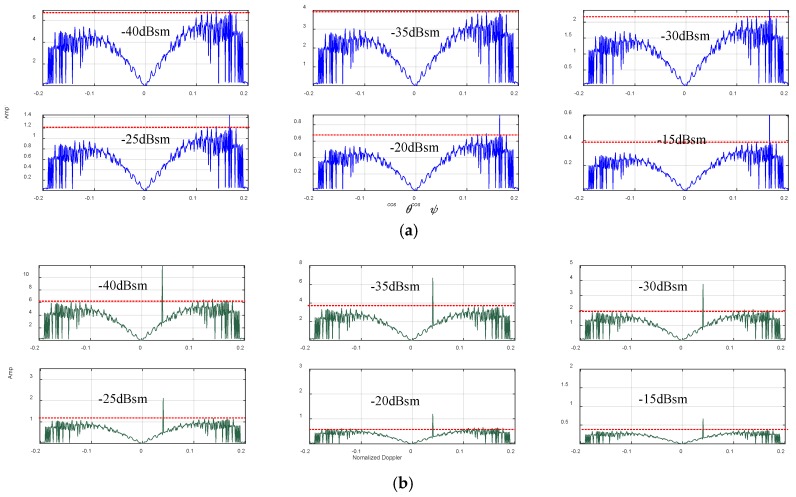
Six object detections by LCMV filtering in (**a**) azimuth; (**b**) doppler under −60dBm noise environment.

Aiming at qualitative analysis, we consider the six objects at 25th 0.5 × 0.5 m cell in 97th stripe (cosθcosψ=0.164). Furthermore, we define the normalized Doppler as $f_{dt}/f_{r}=0.04$. Simulation results in azimuth and Doppler are respectively given in Figure 11.

Disturbed by −60 dBm noise, the simulations in Figure 11a demonstrate that false alarms are produced and the interference effective azimuth indication occurs when a target RCS is smaller than −30 dBsm. Meanwhile, we have also noticed that false alarms, generated by non-homogeneous scattering within radar coverage, exist all over the space-time plane especially in the azimuth domain. But the space-time filtering works well to all six targets in Doppler very clearly. Theoretically, motive objects are distinguished from the scattering cells in Doppler, which benefit the SAR system. In practical operation, a lower false alarm rate could be obtained by setting a threshold decision coefficient according to the characteristics and the distribution of clutter, which will be validated in future work.

**Figure 12:**
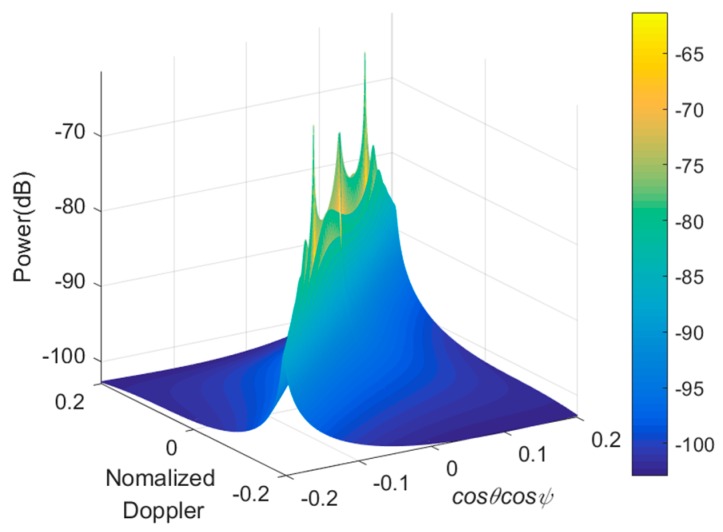
MV spectrum of clutter estimated by samples of 20th to 50th range gates, where refers to both runway and lawn surface.

**Figure 13:**
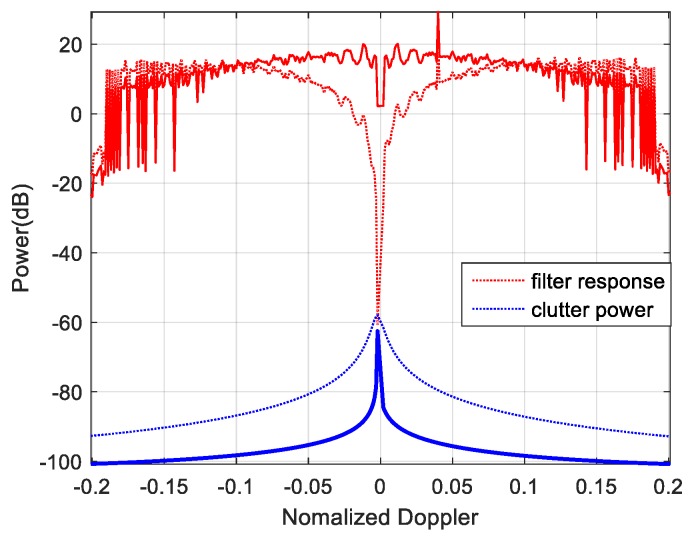
Comparison between the proposed method and traditional processing aiming at a −20 dBsm object, where the solid lines indicate poor clutter suppression under −60 dBm noise.

In order to illustrate that the proposed method performs better than state-of-the-arts, samples from different range gates are utilized for filtering in the space-time domain. The number of range gates are expressed as $\left\lceil \left(R_{\max}-R_{\min}\right)/\Delta R\rceil =2B\lceil \left(R_{\max}-R_{\min}\right)/c\right\rceil$ where ⌈⋅⌉ denotes rounding-up. Thus, there are 439 range gates covered by the SAR according to the parameter setting in Table 3. Figure 12 illustrated the MV spectrum brought by the error of clutter covariance matrix estimation, employing insufficient IID echo from some range gates. As a result, traditional estimation in Figure 13 cannot provide satisfactory clutter suppression when compared with the presented STAP. Obviously, estimation errors directly lead to unnecessary suppression on the space-time plane rather than the main clutter field, which always manifests as decreased SCNR of the filter output.

## 6. Conclusions

STAP methods for moving FOD detection deserves more attention for many compelling advantages such as lower cost, more flexibility and higher resolution. However, the performance of the conventional statistical STAP meets great degradation under nonhomogeneous samples or environment. This paper proposed a clutter-analysis-based Space-time Adaptive Processing (STAP) method in order to obtain effective clutter suppression and moving FOD indication, under inhomogeneous clutter background. We first divided the radar coverage into equal scattering cells in the rectangular coordinates system rather than the polar ones. We then measured normalized RCSs within the X-band and employed the acquired results to modify the parameters of traditional models. Finally, we described the clutter expressions as responses of the scattering cells in the space and time domain to obtain the theoretical clutter covariance. Experimental results at 10 GHz indicated that FODs with a reflection higher than −30 dBsm could be effectively detected by a LCMV filter in azimuth when the noise was −60 dBm. It was also validated to indicate a −40 dBsm target in Doppler. The approach could obtain effective clutter suppression 60 dB deeper than the training-sample-coupled STAP under the same conditions.

Nevertheless, key problems confronted in real-world applications are presented for this STAP technique, which include false alarm effect, influence of spatial errors, and huge computational cost with exact cell division. Moreover, we also plan to investigate its performance under non-ideal conditions such as the presence of SAR yaw, steering vector mismatch, and complex terrains. Therefore, it is of great value to develop robust algorithms. We also realize that thinner item detection disturbed by strong interferences is the most difficult task in meeting practical demands. Future work will include testing on an airport runway to measure practicability and accuracy.

## Figures and Tables

**Table 1:** Parameter choices of Kulemin model [41,47].

Surface Type	Concrete Runway			Lawn (Height ≤ 0.5 m)		
**Parameter**	$A_{1}$	$A_{2}$	$A_{3}$	$A_{1}$	$A_{2}$	$A_{3}$
**Value**	−49	32	20	−21	10	6

**Table 2:** The condition setting of backscatter coefficient measurement.

Data Number	Date	Rf Frequency	Frequency Step	Azimuth	Azimuth Step	Grazing Angle
**Dataset 1**	16 September 2014	10 GHz	\	−180–180 deg	0.1 deg	5/10/15 deg
**Dataset 2**	16 December 2014	8–12 GHz	10 MHz			

**Table 3:** Parameter setting of simulations.

	Parameter	Symbol	Value	Parameter	Symbol	Value
SAR	number of pulses in a CPI	K	3	number of antenna elements	N	4
	PRI	$T_{r}$	0.1 ms	ULA spacing	d	0.5 λ
	beam width in azimuth	$\theta_{B}$	20°	beam width in downwards	α	2.4°
	transmit power	$P_{t}$	100 mW	antenna gain	G	48 dBi
	scale	β	1	\	\	\
Scene knowledge	distance	$R_{1}$	50 m	platform height	h	2 m
	runway width	$R_{2}$	50 m	\	\	\

## Appendix A

In detail, we rewrite Equation (5) at first:

(A1) $$\begin{array}{l} \xi^{0}\left(\mathrm{dB}\right)=A_{1}+A_{2}log\psi -A_{2}log20+A_{3}logf_{c}-A_{3}log10\\ \;\;\;\;\;=A_{2}log\psi +\left(A_{1}-A_{2}log20+A_{3}logf_{c}-A_{3}log10\right)=A_{2}x+b \end{array}$$

If Num measurements are taken, Equation (A1) can be expressed in the vector form as

(A2) $$Y=A_{2}X+b$$

where $Y={\left[\xi_{1}^{0},\xi_{2}^{0},\cdots ,\xi_{Num}^{0}\right]}^{T}$ is collected in case of $X={\left[\log\psi_{1},\log\psi_{2},,\cdots ,\log\psi_{Num}\right]}^{T}$. Notice that $b=A_{1}-A_{2}log20+A_{3}logf_{c}-A_{3}log10$ is constant thus $b={\underset{Num}{\underbrace{\left[b,b,\cdots ,b\right]}}}^{T}$. Two functions are introduced as following:

(A3) $$\Phi_{1}\left(X\right)={\left[\phi_{1}\left(x_{1}\right),\phi_{1}\left(x_{2}\right),\cdots ,\phi_{1}\left(x_{Num}\right)\right]}^{T}={\underset{Num}{\underbrace{\left[1,1,\cdots ,1\right]}}}^{T}\;\Phi_{2}\left(X\right)={\left[\phi_{2}\left(x_{1}\right),\phi_{2}\left(x_{2}\right),\cdots ,\phi_{2}\left(x_{Num}\right)\right]}^{T}={\left[x_{1},x_{2},\cdots ,x_{Num}\right]}^{T}\;={\left[\log\psi_{1},\log\psi_{2},,\cdots ,\log\psi_{Num}\right]}^{T}$$

then calculate the inner products among these two items and Y and put them into Equation (8):

(A4) $$\{\begin{array}{c} \left(\Phi_{1},Y\right)=\left(\Phi_{1},\Phi_{2}\right)A_{2}+\left(\Phi_{1},\Phi_{1}\right)b\\ \left(\Phi_{2},Y\right)=\left(\Phi_{2},\Phi_{2}\right)A_{2}+\left(\Phi_{1},\Phi_{2}\right)b \end{array}$$

We express Equation (A4) in form of matrix as

(A5) $$\left(\begin{array}{cc} \langle \Phi_{1},\Phi_{2}\rangle & \langle \Phi_{1},\Phi_{2}\rangle \\ \langle \Phi_{2},\Phi_{2}\rangle & \langle \Phi_{1},\Phi_{2}\rangle \end{array}\right)\left(\begin{array}{c} A_{2}\\ b \end{array}\right)=\left(\begin{array}{c} \langle \Phi_{1},Y\rangle \\ \langle \Phi_{2},Y\rangle \end{array}\right)$$

Hence both $A_{2}$ and b can be solved based on Equation (A5)

(A6) $$\left(\begin{array}{c} A_{2}\\ b \end{array}\right)={\left(\begin{array}{cc} \langle \Phi_{1},\Phi_{2}\rangle & \langle \Phi_{1},\Phi_{2}\rangle \\ \langle \Phi_{2},\Phi_{2}\rangle & \langle \Phi_{1},\Phi_{2}\rangle \end{array}\right)}^{-1}\left(\begin{array}{c} \langle \Phi_{1},Y\rangle \\ \langle \Phi_{2},Y\rangle \end{array}\right)$$

then we could acquire backscattering coefficients in low grazing angles according to the modified model $\xi^{0}\left(\mathrm{dB}\right)=A_{2}log\psi +b$.
